# Supplementary material for: Does a Higher Protein Diet Promote Satiety and Weight Loss Independent of Carbohydrate Content? An 8-Week Low-Energy Diet (LED) Intervention
Source: Nutrients. 2022 Jan 26;14(3):538. doi: 10.3390/nu14030538 (PMC8838013; doi:10.3390/nu14030538)
Supplement: Supplementary file 1 [file nutrients-14-00538-s001.zip › nutrients-1527278-supplementary.pdf]

Table S1. The effect of dietary groups on anthropometry, body composition, serum lipid, glucose, glucoregulatory peptides, gut peptides, and amino acids measured at Week 0, 4 and 8.

	Week	HPLC	HPNC	NPLC	NPNC	*p*-value		
						Protein× Week	CHO× Week	Protein× CHO*Week
Anthropometry								
BMI (kg/m^2)	0	35.5 ± 0.3	35.4 ± 0.3	35.4 ± 0.3	35.4 ± 0.3	0.738	0.440	0.927
	4	33.4 ± 0.3	33.9 ± 0.3	33.4 ± 0.3	33.7 ± 0.4			
	8	32.5 ± 0.5	32.3 ± 0.3	33.1 ± 0.3	32.7 ± 0.4			
Waist circumference	0	100.7 ± 0.9	100.8 ± 0.9	100.7 ± 0.9	100.6 ± 0.9	0.924	0.258	0.542
(cm)	4	96.1 ± 1.0	97.0 ± 0.9	95.5 ± 0.9	98.6 ± 0.9			
	8	93.4 ± 1.3	93.4 ± 1.1	92.9 ± 1.1	94.5 ± 1.0			
WHR	0	0.84 ± 0.01	0.84 ± 0.01	0.84 ± 0.01	0.84 ± 0.01	0.973	0.293	0.453
	4	0.83 ± 0.01	0.84 ± 0.01	0.82 ± 0.01	0.86 ± 0.01			
	8	0.82 ± 0.01	0.82 ± 0.01	0.81 ± 0.01	0.84 ± 0.01			
SBP (mmHg)	0	117.2 ± 1.3	116.0 ± 1.4	115.9 ± 1.3	116.2 ± 1.4	0.864	0.487	0.478
	4	110.7 ± 1.4	113.5 ± 1.4	112.4 ± 1.3	112.7 ± 1.5			
	8	108.4 ± 1.4	108.9 ± 1.6	107.9 ± 1.5	110.4 ± 1.7			
DBP (mmHg)	0	63.5 ± 0.7	62.3 ± 0.7	62.2 ± 0.7	62.9 ± 0.7	0.297	0.074	0.309
	4	60.4 ± 0.7	59.8 ± 0.8	60.6 ± 0.7	59.4 ± 0.8			
	8	57.8 ± 0.8	59.8 ± 0.9	59.3 ± 0.8	60.4 ± 0.9			
Body composition								
FM (kg)	0	44.4 ± 0.5	44.4 ± 0.5	44.4 ± 0.5	44.4 ± 0.5	0.929	0.781	0.783
	8	38.4 ± 1.0	39.0 ± 0.6	38.6 ± 0.6	38.7 ± 1.2			
FM (% body mass)	0	46.8 ± 0.6	46.8 ± 0.6	46.8 ± 0.6	46.8 ± 0.6	0.728	0.940	0.877
	8	43.8 ± 0.9	43.9 ± 0.8	44.4 ± 0.7	44.1 ± 1.4			
FFM (kg)	0	50.1 ± 0.8	50.1 ± 0.8	50.0 ± 0.8	50.1 ± 0.8	0.657	0.621	0.998
	8	48.6 ± 1.1	49.4 ±1.1	47.8 ± 0.9	48.7 ± 1.7			
Serum lipid								
Total-C (mM)	0	5.26 ± 0.13	5.25 ± 0.14	5.33 ±0.13	5.30 ± 0.13	0.317	0.438	0.393
	8	4.26 ± 0.15	4.63 ± 0.19	4.30 ± 0.14	4.29 ± 0.21			
HDL-C (mM)	0	1.43 ± 0.04	1.44 ± 0.04	1.42 ± 0.04	1.45 ± 0.04	0.542	0.124	0.968
	8	1.28 ± 0.04	1.21 ± 0.05	1.22 ± 0.04	1.18 ± 0.04			
Total:HDL-C (mM)	0	4.01 ± 0.21	3.91 ± 0.21	4.05 ± 0.20	3.88 ± 0.21	0.686	0.134	0.549
	8	3.37 ± 0.22	3.97 ± 0.29	3.75 ± 0.22	3.87 ± 0.24			
LDL-C (mM)	0	3.43 ± 0.10	3.42 ± 0.10	3.49 ± 0.10	3.46 ± 0.10	0.269	0.444	0.225
	8	2.77 ± 0.10	3.08 ± 0.11	2.82 ± 0.10	2.71 ± 0.18			
Trig (mM)	0	1.66 ± 0.16	1.40 ± 0.17	1.46 ± 0.16	1.56 ± 0.16	0.922	0.460	0.057
	8	0.80 ± 0.17	1.17 ± 0.17	1.07 ± 0.16	0.90 ± 0.17			
NEFA (mM)	0	0.47 ± 0.03	0.49 ± 0.03	0.51 ± 0.03	0.50 ± 0.03	0.207	0.788	0.229

	8	0.71 ± 0.04	0.65 ± 0.04	0.63 ± 0.04	0.66 ± 0.05			
Glucose and glucoregulatory peptides								
Glucose (mM)	0	5.65 ± 0.10	5.65 ± 0.10	5.77 ± 0.10	5.62 ± 0.10	0.746	0.547	0.461
	4	5.34 ± 0.10	5.33 ± 0.10	5.33 ± 0.10	5.46 ± 0.11			
	8	5.25 ± 0.12	5.42 ± 0.11	5.31 ± 0.11	5.25 ± 0.17			
Insulin (pg/mL)	0	684.5 (541.0 – 866.0)	699.6 (533.0 – 885.1)	738.9 (588.6 – 927.5)	775.7 (615.6 – 977.6)	0.754	0.634	0.503
	4	502.6 (502.6 – 651.0)	599.9 (471.4 – 763.5)	565.5 (448.7 – 712.6)	531.4 (396.8 – 711.6)			
	8	533.1 (420.5 – 675.8)	615.2 (477.7 – 792.1)	534.8 (409.7 – 698.2)	629.2 (473.6 – 836.1)			
Glucagon (pg/mL)	0	47.6 (41.4 – 54.7)	48.9 (42.4 – 56.5)	47.7 (41.5 – 54.8)	51.3 (44.6 – 59.0)	**0.050**	0.490	0.767
	4	45.6 (39.7 – 52.4)	49.1 (42.4 – 57.0)	33.7 (29.1 – 39.1)	42.9 (36.6 – 50.4)			
	8	41.0 (34.6 – 48.6)	44.1 (37.4 – 51.9)	36.1 (30.8 – 42.3)	40.8 (34.9 – 47.7)			
GIP (pg/mL)	0	45.2 (37.8 – 53.9)	42.6 (35.5 – 51.1)	49.1 (41.2 – 58.6)	45.1 (37.7 – 54.0)	0.282	0.323	0.941
	4	39.7 (33.3 – 47.5)	41.8 (34.8 – 50.3)	37.2 (31.2 – 44.4)	36.5 (29.4 – 45.2)			
	8	38.9 (32.5 – 46.6)	43.3 (35.1 – 53.5)	35.7 (29.5 – 43.2)	40.2 (32.8 – 49.4)			
Gut peptides								
GLP-1 (pg/mL)	0	172.7 (151.7 – 196.7)	169.7 (148.5 – 193.9)	170.9 (150.2 – 194.5)	178.1 (156.2 – 203.1)	0.176	0.675	0.600
	4	181.6 (157.5 – 209.3)	177.8 (152.2 – 207.8)	140.0 (121.9 – 160.8)	167.8 (144.5 – 195.0)			
	8	150.4 (129.1 – 175.4)	166.7 (138.9 – 200.0)	133.8 (114.1 – 157.1)	147.5 (127.6 – 170.5)			
PYY (pg/mL)	0	34.9 (29.4 – 41.5)	39.4 (33.0 – 47.1)	39.3 (33.1 – 46.6)	36.8 (30.9 – 43.8)	0.806	0.963	0.785
	4	33.8 (27.2 – 42.1)	33.4 (27.4 – 40.7)	32.1 (26.9 – 38.3)	32.2 (26.9 – 38.6)			
	8	33.5 (27.3 – 41.1)	35.6 (28.6 – 44.2)	36.3 (30.3 – 43.4)	34.7 (25.9 – 46.4)			
Branched-chain amino acids								
Leucine (μM)	0	122.5 ± 3.4	120.5 ± 3.5	121.3 ± 3.4	119.6 ± 3.5	0.974	0.545	0.673
	4	118.6 ± 4.1	122.7 ± 3.6	117.2 ± 3.7	120.1 ± 3.8			
	8	125.3 ± 3.5	121.5 ± 3.6	119.7 ± 4.0	123.2 ± 3.9			
Isoleucine (μM)	0	66.0 ± 2.4	66.5 ± 2.4	64.8 ± 2.4	63.3 ± 2.4	0.556	0.702	0.590
	4	64.0 ± 2.5	67.1 ± 2.5	66.5 ± 2.5	67.8 ± 3.2			
	8	67.9 ± 2.5	65.0 ± 2.8	65.6 ± 2.5	67.6 ± 3.2			
Valine (μM)	0	238.8 ± 6.7	234.5 ± 6.9	238.0 ± 6.7	233.9 ± 6.8	0.650	0.514	0.888
	4	239.3 ± 7.6	238.3 ± 7.1	227.7 ± 7.0	234.3 ± 7.4			
	8	249.9 ± 7.0	238.0 ± 7.2	235.9 ± 7.0	233.1 ± 7.8			
Other essential amino acids								
Phenylalanine (μM)	0	55.6 ± 1.3	56.8 ± 1.4	55.4 ± 1.3	55.5 ± 1.3	0.829	0.882	0.894
	4	54.0 ± 1.7	54.7 ± 1.4	51.9 ± 1.4	52.7 ± 1.7			

	8	53.1 ± 1.4	53.9 ± 1.6	52.5 ± 1.5	51.3 ± 1.9			
Methionine (μM)	0	28.0 ± 0.9	28.4 ± 0.9	28.2 ± 0.9	27.1 ± 0.9	0.524	0.701	0.135
	4	26.9 ± 1.0	24.8 ± 0.9	26.2 ± 0.9	27.5 ± 1.1			
	8	25.4 ± 1.1	25.8 ± 1.1	25.5 ± 1.0	26.3 ± 1.0			
Lysine (μM)	0	84.6 ± 2.4	82.3 ± 2.4	83.8 ± 2.4	84.3 ± 2.4	0.338	0.259	0.199
	4	81.7 ± 2.6	87.5 ± 2.6	83.4 ± 2.5	84.1 ± 2.9			
	8	77.7 ± 2.6	87.7 ± 2.5	78.0 ± 2.4	78.4 ± 2.8			
Histidine (μM)	0	52.8 ± 2.3	55.2 ± 2.4	53.1 ± 2.3	54.7 ± 2.4	0.665	0.867	0.737
	4	49.6 ± 2.7	56.7 ± 2.4	50.9 ± 2.4	51.9 ± 2.9			
	8	53.2 ± 3.1	57.3 ± 2.6	50.9 ± 2.4	52.6 ± 2.8			
Threonine (μM)	0	110.7 ± 3.7	114.4 ± 3.8	110.6 ± 3.7	111.1 ± 3.7	0.936	0.998	0.312
	4	109.4 ± 4.3	110.6 ± 4.0	109.0 ± 4.0	111.4 ± 4.0			
	8	99.2 ± 3.8	108.3 ± 4.6	105.2 ± 4.1	99.8 ± 4.7			
Tryptophan (μM)	0	39.6 ± 1.4	40.8 ± 1.5	38.9 ± 1.4	39.5 ± 1.5	0.941	0.862	0.852
	4	39.1 ± 1.5	38.6 ± 1.5	37.2 ± 1.5	37.1 ± 2.2			
	8	36.1 ± 1.8	37.6 ± 1.7	35.9 ± 1.7	35.3 ± 2.2			
Non-essential amino acids								
Glycine (μM)	0	277.1 ± 12.1	275.9 ± 12.5	272.0 ± 12.2	276.8 ± 12.3	0.622	0.647	0.878
	4	342.7 ± 12.7	331.5 ± 13.3	344.7 ± 13.2	323.7 ± 17.9			
	8	265.0 ± 14.7	257.5 ± 14.7	273.9 ± 14.5	273.5 ± 14.1			
Aspartic acid (μM)	0	5.8 ± 0.5	6.0 ± 0.5	5.3 ± 0.5	4.9 ± 0.5	0.444	0.569	0.979
	4	3.6 ± 0.5	4.0 ± 0.5	3.9 ± 0.5	4.1 ± 0.5			
	8	3.8 ± 0.6	4.7 ± 0.5	3.7 ± 0.6	4.2 ± 0.6			
Asparagine (μM)	0	48.1 ± 1.2	48.8 ± 1.3	48.2 ± 1.2	46.8 ± 1.2	0.497	0.506	0.956
	4	47.8 ± 1.3	46.8 ± 1.3	49.8 ± 1.2	47.1 ± 1.6			
	8	45.0 ± 1.3	46.7 ± 1.4	45.9 ± 1.3	44.8 ± 1.5			
Glutamic acid (μM)	0	59.5 ± 3.7	53.9 ± 3.8	54.2 ± 3.7	52.3 ± 3.7	0.719	0.068	0.665
	4	37.7 ± 4.3	46.2 ± 3.9	38.3 ± 3.6	47.5 ± 3.9			
	8	40.2 ± 4.2	46.8 ± 4.1	41.9 ± 3.9	42.7 ± 4.0			
Glutamine (μM)	0	529.4 ± 9.9	541.1 ± 10.2	537.5 ± 9.9	530.8 ± 10.0	0.525	0.992	0.971
	4	518.2 ± 10.5	527.0 ± 10.4	537.7 ± 11.1	535.2 ± 13.3			
	8	517.8 ± 11.8	530.5 ± 12.4	527.4 ± 11.1	523.6 ± 13.0			
Arginine (μM)	0	74.5 ± 3.4	78.0 ± 3.5	75.1 ± 3.4	77.1 ± 3.4	0.800	0.855	0.333
	4	70.6 ± 3.5	84.3 ± 3.6	75.5 ± 3.6	72.6 ± 4.2			
	8	72.2 ± 3.4	79.6 ± 3.8	73.1 ± 3.5	72.2 ± 4.3			
Alanine (μM)	0	401.5 ± 10.4	415.7 ± 10.8	411.7 ± 10.4	389.4 ± 10.6	0.971	0.319	0.140
	4	357.8 ± 11.5	336.2 ± 11.9	340.1 ± 11.0	346.4 ± 13.7			
	8	324.1 ± 11.3	349.2 ± 11.2	328.0 ± 11.3	336.3 ± 13.2			
Serine (μM)	0	105.9 ± 3.4	104.0 ± 3.5	106.6 ± 3.4	105.6 ± 3.4	0.839	0.912	0.783
	4	118.7 ± 3.5	112.7 ± 3.8	116.2 ± 3.7	117.2 ± 3.9			

	8	121.3 ± 4.2	120.5 ± 3.9	119.3 ± 3.9	119.5 ± 4.3			
Tyrosine (μM)	0	66.4 ± 1.8	68.2 ± 1.8	67.2 ± 1.8	66.0 ± 1.8	0.984	0.887	0.680
	4	59.8 ± 1.8	59.6 ± 1.8	58.7 ± 1.8	59.2 ± 1.9			
	8	57.0 ± 2.0	60.3 ± 2.0	57.7 ± 1.8	57.2 ± 2.6			
Proline (μM)	0	208.6 ± 7.0	217.5 ± 7.3	207.6 ± 7.0	198.5 ± 7.2	0.670	0.541	0.217
	4	195.4 ± 8.8	184.4 ± 8.0	186.6 ± 7.2	195.8 ± 9.6			
	8	165.7 ± 9.2	178.7 ± 9.6	162.1 ± 7.5	169.5 ± 8.7			
Non-proteogenic amino acids								
Hydroxyproline (μM)	0	14.7 ± 0.9	14.6 ± 0.9	13.2 ± 0.9	12.8 ± 0.9	0.740	0.246	0.711
	4	11.2 ± 1.0	14.2 ± 1.0	11.0 ± 0.9	11.7 ± 1.1			
	8	9.5 ± 1.1	12.3 ± 1.0	10.0 ± 1.0	10.6 ± 1.0			
Taurine (μM)	0	131.1 ± 9.4	119.6 ± 9.6	106.2 ± 9.3	101.8 ± 9.4	**0.045**	0.471	0.382
	4	108.7 ± 10.0	109.3 ± 10.0	119.1 ± 9.7	126.3 ± 10.0			
	8	85.2 ± 9.4	106.2 ± 10.0	104.4 ± 9.4	99.4 ± 11.2			
Citrulline (μM)	0	29.0 ± 0.7	29.5 ± 0.7	29.5 ± 0.7	29.6 ± 0.7	0.863	0.512	0.297
	4	26.4 ± 0.8	28.0 ± 0.7	27.7 ± 0.7	26.0 ± 0.9			
	8	26.9 ± 0.9	27.6 ± 0.8	28.4 ± 0.7	25.9 ± 1.1			
Ornithine (μM)	0	38.7 ± 1.7	38.3 ± 1.8	39.7 ± 1.7	39.0 ± 1.7	0.781	0.172	0.568
	4	32.6 ± 1.8	35.2 ± 1.8	37.0 ± 1.8	35.5 ± 2.5			
	8	32.8 ± 2.0	39.5 ± 2.2	35.6 ± 1.9	37.0 ± 2.9			

Data reported as estimated marginal means ± SEM or 95% CI following linear mixed model using multiple imputed data. All analyses were statistically adjusted for baseline measurements. BMI, body mass index; WHR, waist-hip ratio; SBP, systolic blood pressure; DBP, diastolic blood pressure; FM, fat mass; FFM, fat free mass; Total-C, total cholesterol; HDL-C, high density lipoprotein cholesterol; Total: HDL-C, Total-to-HDL cholesterol ratio; LDL-C, low density lipoprotein cholesterol; Trig, triglycerides; NEFA, non-esterified fatty acids; GIP, gastric inhibitory polypeptide; GLP-1, glucagon-like peptide-1; PYY, peptide YY.

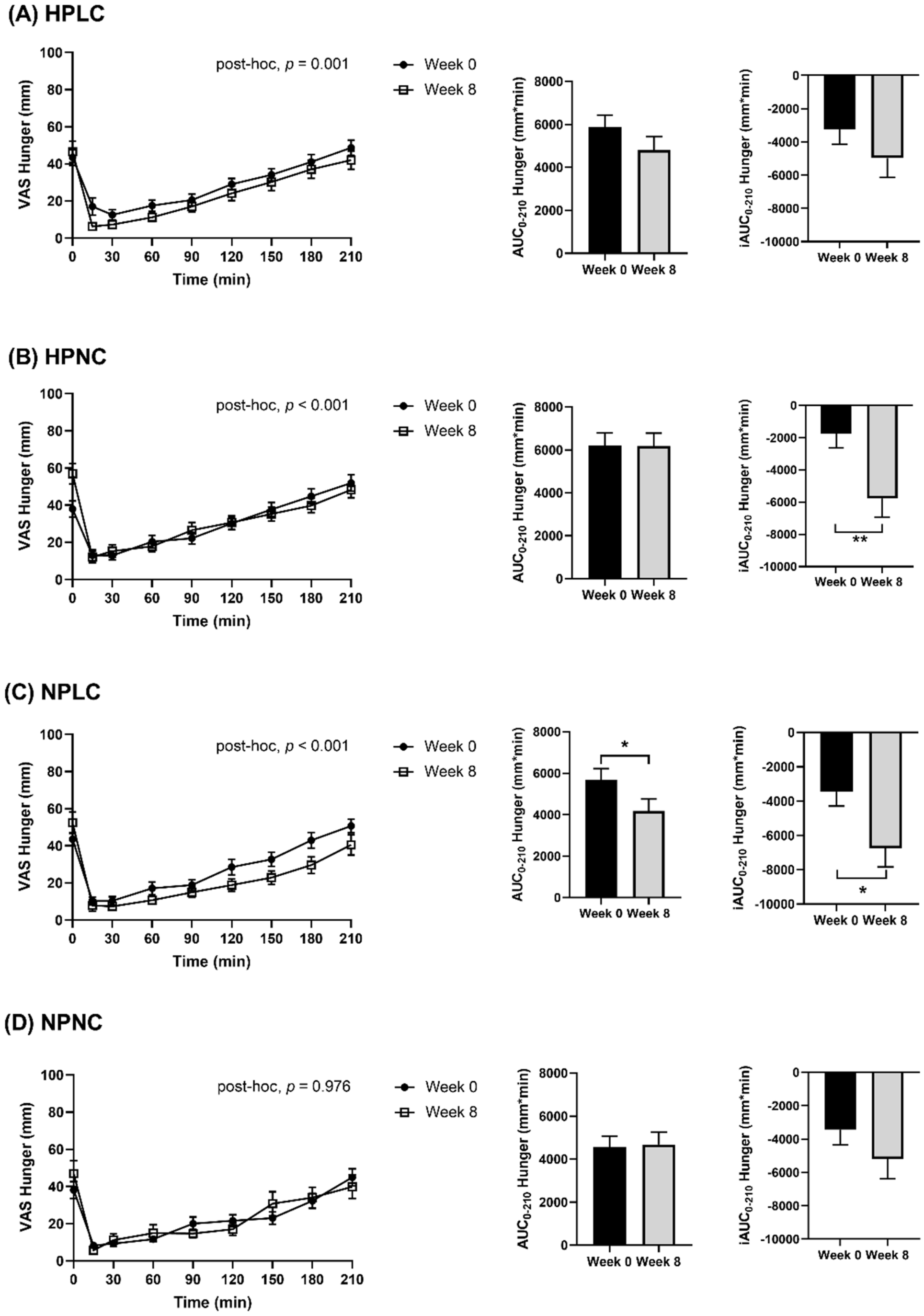

Figure S1. Observed mean (±SEM) VAS Hunger for t = 0 – 210 min, AUC$_{0-210}$ and iAUC$_{0-210}$ following (**A**) HPLC, (**B**) HPNC, (**C**) NPLC, and (**D**) NPNC. Number of participants completed appetite VAS at Week 0 and Week 8 following HPLC were n = 31 and 23, HPNC were n = 29 and 23, NPLC were n = 31 and 27, and NPNC were n = 30 and 18. Observed repeated measures data (t = 0 – 210 min) were analysed using a linear mixed model, adjusted for differences in fasted ratings (t = 0 min). Post-hoc analysis shows the statistically significant difference in postprandial appetite responses between Week 0 and Week 8. AUC$_{0-210}$ and iAUC$_{0-210}$ between Week 0 and Week 8 were compared using mixed model ANOVA, *$p < 0.05$, **$p < 0.01$.

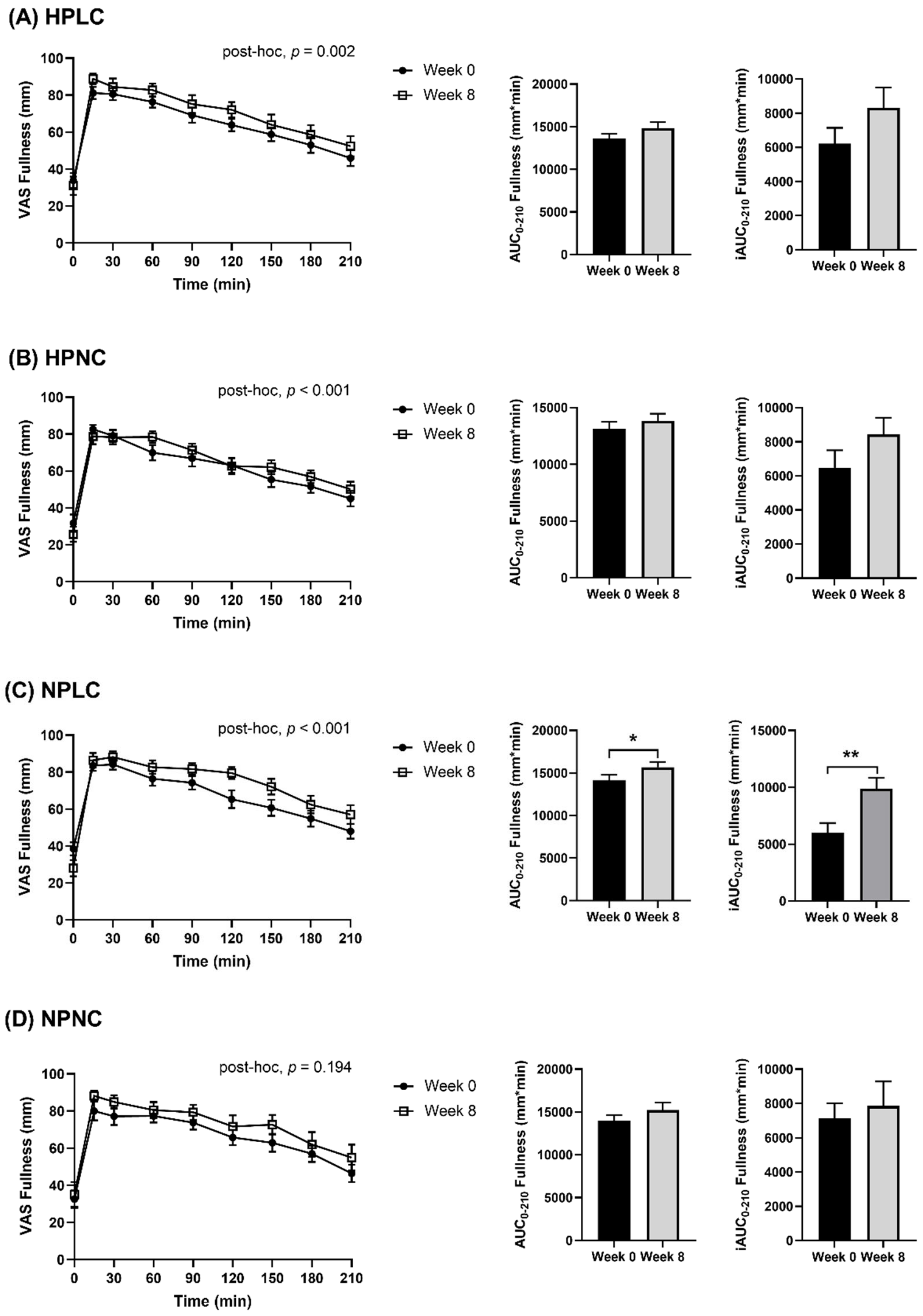

Figure S2. Observed mean (±SEM) VAS Fullness for t = 0 – 210 min, AUC$_{0\text{-}210}$ and iAUC$_{0\text{-}210}$ following (**A**) HPLC, (**B**) HPNC, (**C**) NPLC, and (**D**) NPNC. Number of participants completed appetite VAS at Week 0 and Week 8 following HPLC were n = 31 and 23, HPNC were n = 29 and 23, NPLC were n = 31 and 27, and NPNC were n = 30 and 18. Observed repeated measures data (t = 0 – 210 min) were analysed using a linear mixed model, adjusted for differences in fasted ratings (t = 0 min). Post-hoc analysis shows the statistically significant difference in postprandial appetite responses between Week 0 and Week 8. AUC$_{0\text{-}210}$ and iAUC$_{0\text{-}210}$ between Week 0 and Week 8 were compared using mixed model ANOVA, *$p < 0.05$, **$p < 0.01$.

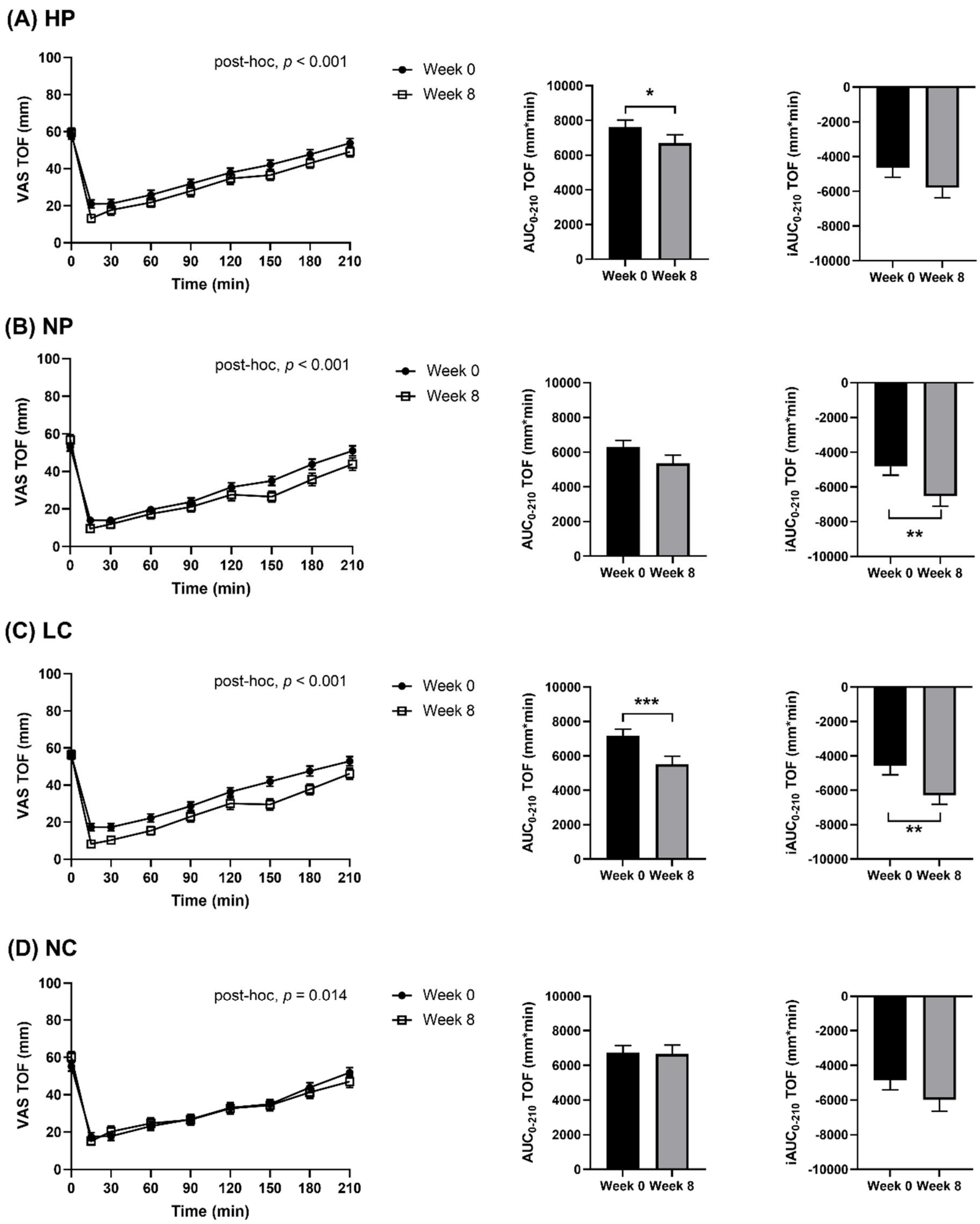

Figure S3. Observed mean (±SEM) VAS TOF for t = 0 – 210 min, AUC_{0-210} and $iAUC_{0-210}$ following (**A**) HP, (**B**) NP, (**C**) LC, and (**D**) NC. Number of participants completed appetite VAS at Week 0 and Week 8 following HP were n = 60 and 46, NP were n = 61 and 45, LC were n = 62 and 50, and NC were n = 59 and 41. Observed repeated measures data (t = 0 – 210 min) were analysed using a linear mixed model, adjusted for differences in fasted ratings (t = 0 min). Post-hoc analysis shows the statistically significant difference in postprandial appetite responses between Week 0 and Week 8. AUC_{0-210} and $iAUC_{0-210}$ between Week 0 and Week 8 were compared using mixed model ANOVA, *$p < 0.05$, **$p < 0.01$. TOF, ***$p < 0.001$, Thoughts of Food.

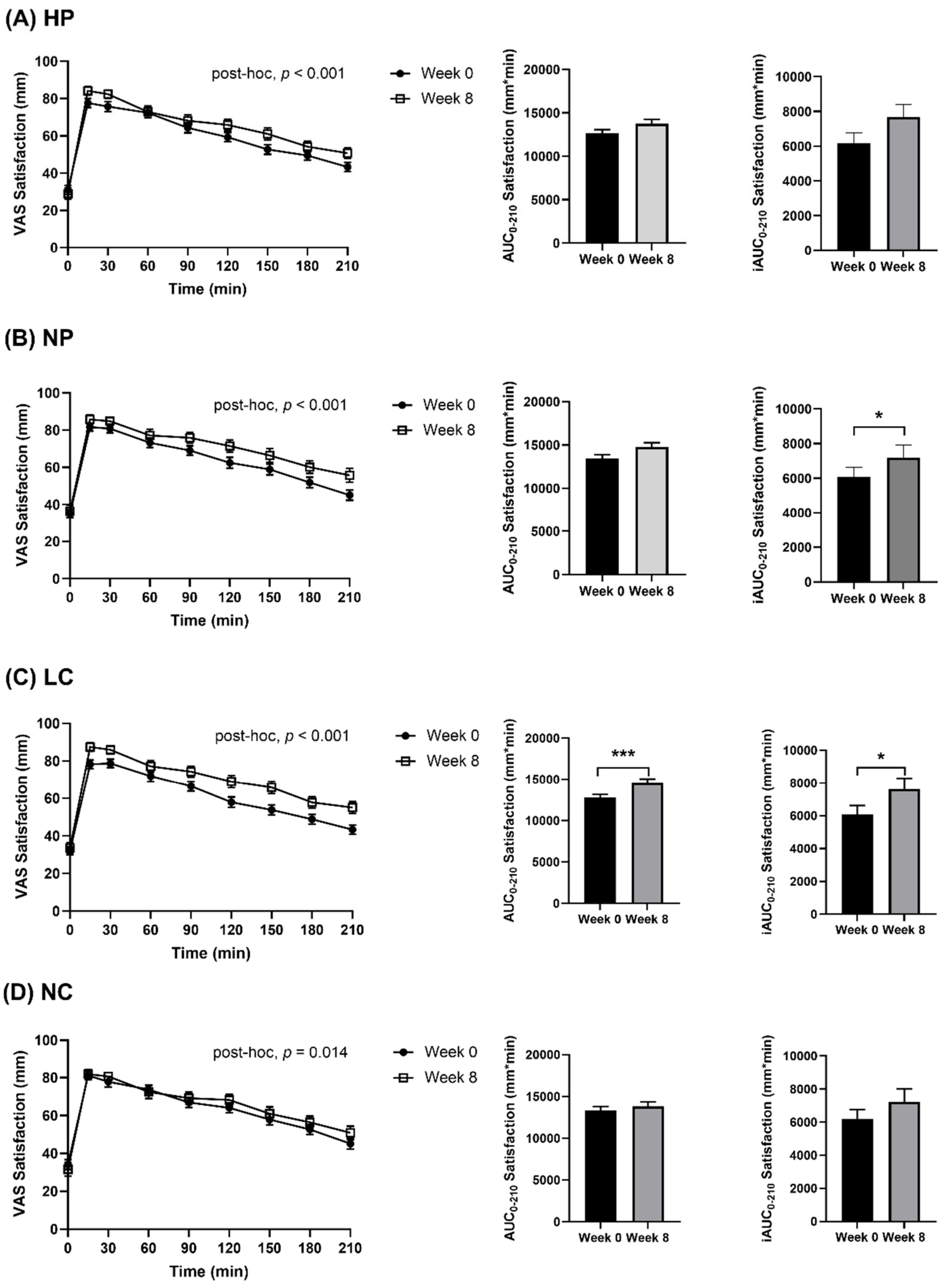

Figure S4. Observed mean (±SEM) VAS Satisfaction for t = 0 – 210 min, $AUC_{0\text{-}210}$ and $iAUC_{0\text{-}210}$ following (**A**) HP, (**B**) NP, (C) LC, and (D) NC. Number of participants completed appetite VAS at Week 0 and Week 8 following HP were $n = 60$ and 46, NP were $n = 61$ and 45, LC were $n = 62$ and 50, and NC were $n = 59$ and 41. Observed repeated measures data (t = 0 – 210 min) were analysed using a linear mixed model, adjusted for differences in fasted ratings (t = 0 min). Post-hoc analysis shows the statistically significant difference in postprandial appetite responses between Week 0 and Week 8. $AUC_{0\text{-}210}$ and $iAUC_{0\text{-}210}$ between Week 0 and Week 8 were compared using mixed model ANOVA, $^{*}p < 0.05$, $^{***}p < 0.001$.

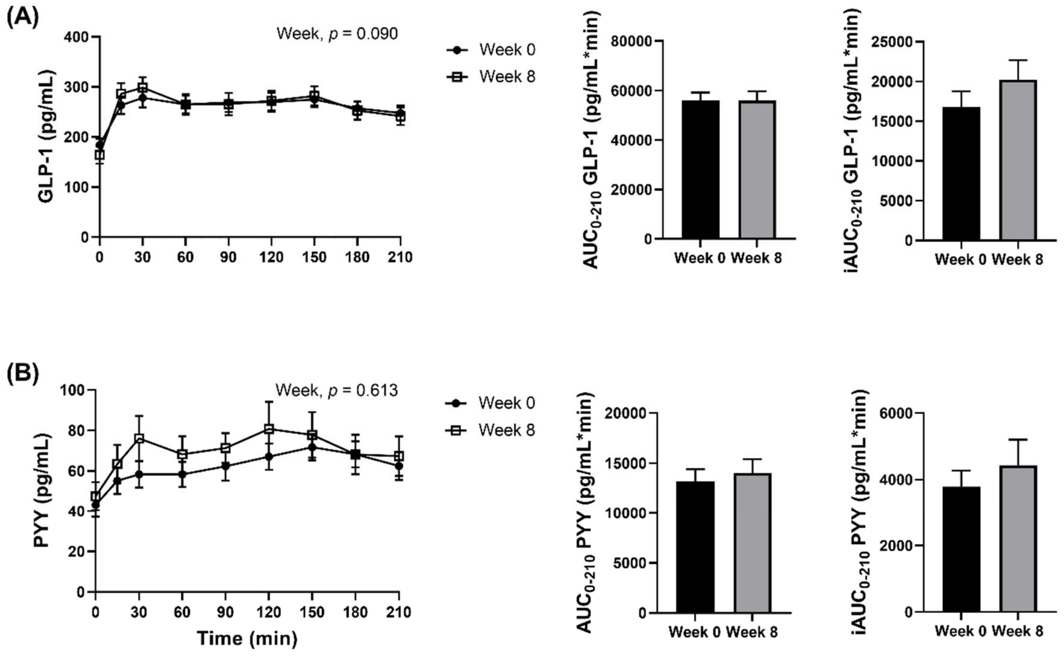

Figure S5. Observed mean (±SEM) concentrations of (**A**) GLP-1 and (**B**) PYY for t = 0 – 210 min, $AUC_{0\text{-}210}$, and $iAUC_{0\text{-}210}$, presented as all dietary groups combined. Number of participants completed the repeated blood samplings procedure at Week 0 and Week 8 were n = 42 and n = 32, respectively. Observed cases data were analysed using a linear mixed model, adjusted for differences in fasted concentrations (t = 0 min). $AUC_{0\text{-}210}$ and $iAUC_{0\text{-}210}$ between Week 0 and Week 8 were compared using mixed model ANOVA.
